# Safety and Efficacy of Tirofiban in Severe Ischemic Stroke Patients Undergoing Mechanical Thrombectomy

**DOI:** 10.3390/jcdd9110408

**Published:** 2022-11-21

**Authors:** Lingxin Cai, Tingting Wang, Aiqing Chen, Chenhan Ling, Jing Xu, Cong Qian, Gao Chen

**Affiliations:** 1 Department of Neurological Surgery, The Second Affiliated Hospital of Zhejiang University School of Medicine, Hangzhou 310003, China; 2 Department of Neurological Surgery, People’s Hospital of Jiashan County, Jiaxing 314199, China

**Keywords:** acute ischemic stroke, mechanical thrombectomy, anterior circulation stroke, tirofiban, outcome

## Abstract

Tirofiban has recently shown encouraging efficacy and safety among acute ischemic stroke (AIS) patients with mechanical thrombectomy (MT). However, the benefits of tirofiban varied among studies depending on the patient’s condition, which was often not well analyzed. This study aimed to identify the characteristics of patients who may obtain the largest benefits from tirofiban. The efficacy endpoint was a favorable outcome defined as a modified Rankin Scale (mRS) score of 0~2 at 90 days. The safety endpoints were intracranial hemorrhage (ICH), symptomatic intracranial hemorrhage (sICH) and mortality at 90 days. Adjusted logistic regression analysis and subgroup analyses were utilized to investigate the factors associated with tirofiban and the outcome. All of 285 patients fit the inclusion criteria. Tirofiban was associated with a higher rate of favorable outcome (aOR 2.033, 95% CI, 1.002~4.123, *p* = 0.043) but not with an increased risk of ICH, sICH or mortality (*p* > 0.05). Moreover, subgroup analyses revealed that tirofiban was associated with favorable outcomes in patients with NIHSS > 14 (aOR 2.778, 95% CI 1.056~7.356, *p* = 0.038) but not in patients with NIHSS ≤ 14 (aOR 1.719, 95% CI 0.646~4.578, *p* = 0.278). No significant heterogeneity was found in the effect of tirofiban across the subgroups of age, sex, ASPECTS, time from onset to puncture, use of t-PA or stroke etiology (*p* for interaction > 0.05). The administration of tirofiban was associated with favorable outcomes in severe ischemic stroke patients, and further studies are needed to confirm this finding.

## 1. Introduction

Mechanical thrombectomy (MT) is an effective therapy for acute ischemic stroke (AIS) patients with large vessel occlusion [1,2]. The goal of MT in AIS patients is timely restoration of flow to the salvageable hypoxic brain tissues, but it faces the challenge of failure to recanalize and the occurrence of reocclusion [3]. Tirofiban is currently widely applied during MT as a rescue therapy implemented to block platelet aggregation and increase the rate of recanalization [4,5]. Recent trials have demonstrated the effect and safety of tirofiban [6,7,8]; however, another study reported that tirofiban did not improve the clinical outcome and increased the risk of intracranial hemorrhage [9]. These contrasting results might be attributed to the differences in inclusion criteria and the different characteristics of patients selected for tirofiban administration in each medical center. Evidence and data on which subgroup may benefit more from tirofiban remain ambiguous. This may result in patients who should benefit from tirofiban not receiving treatment, and patients who are not suitably subjected to a high risk of bleeding from tirofiban.

We hypothesized that the efficacy and safety of tirofiban were modified by patient characteristics and the severity of stroke, and tested this hypothesis in a retrospective cohort study of patients with MT. Adjusted logistic regression analysis and subgroup analysis were utilized to investigate factors related to the association between tirofiban and outcome to evaluate which stratified population benefitted most.

## 2. Materials and Methods

### 2.1. Patient Selection

This is a retrospective analyzing consecutive patients with anterior circulation occlusion who underwent mechanical thrombectomy between January 2016 and February 2022. We included patients based on the following criteria: (1) patients who underwent MT employing second-generation stent-retriever devices (Solitaire AB/FR, Covidien/ev3, Irvine, CA; Trevo Proview, Stryker, CA); (2) patients with occlusion of the anterior circulation large artery defined by digital subtraction angiography (DSA); (3) modified Rankin Scale (mRS) score before the index stroke ≤ 1; and (4) time from onset to puncture (OTP) < 12 h. The exclusion criteria were as follows: (1) intracranial hemorrhage defined in CT scan prior to MT; (2) Severe heart, liver, and renal insufficiency, or severe diabetes (blood glucose level > 22 mmol/L); (3) Coagulation dysfunction such as platelet count 100 × 10^9^/L; (4) Missing case data or imaging examination results or loss to follow-up. Our study protocol was approved by the human ethics committee of The Second Affiliated Hospital of Zhejiang University.

### 2.2. Management of Tirofiban

The decision to administer tirofiban was left to the attending physician, and tirofiban was considered for application in the following situations: (1) rescue treatment with emergency stenting for residual artery stenosis or failed thrombectomy, (2) balloon angioplasty for severe residual stenosis or instant reocclusion, (3) successful mechanical recanalization with ≥3 passes with a stent retriever for presumed endothelial damage or instant, and (4) local new thrombosis or vascular dissection of the responsible vessel and other situations with a high risk of early reocclusion. Tirofiban was continuously given at a speed of 8 µg/kg h after an intravenous bolus of 10 µg/kg if there was no evidence of ICH on immediate head CT after MT. 24 h later, dual antiplatelet therapy was given after ICH was ruled out by another head CT.

### 2.3. Data Compilation

We analyze retrospectively the patient data of demographic, clinical, and laboratory data, including age, sex, stroke etiology, baseline National Institutes of Health Stroke Scale (NIHSS), Alberta Stroke Program Early Computed Tomography Score (ASPECTS), baseline blood systolic (BP), blood glucose, platelets, and comorbid conditions, such as history of smoking, hypertension, diabetes mellitus, hyperlipidemia, atrial fibrillation, and prior antiplatelet usage. The stroke etiology was classified according to the Trial of ORG 10172 in Acute Stroke Treatment (TOAST) criteria: large artery atherosclerosis (LAA), cardioembolism (CE), and undetermined etiology (UE). Procedural variables were extracted, including time from onset to groin puncture (OTP) and recanalization (OTR), t-PA use, and Thrombolysis in Cerebral Infarction (TICI) grading. Substantial reperfusion was defined as a modified Thrombolysis in Cerebral Infarction score of 2b-3.

### 2.4. Assessment Criteria

The primary efficacy endpoint was a favorable outcome defined as a modified Rankin Scale (mRS) score of 0~2 at 90 days. The safety endpoints included intracranial hemorrhage (ICH), symptomatic intracranial hemorrhage (sICH) and mortality at 90 days. sICH was defined as hemorrhage associated with deterioration of neurological function, NIHSS score above baseline of 4 points, or death (according to European Cooperative Acute Stroke Study [ECASS] II criteria) [10]. All patients underwent cranial CT within 12 to 24 h after MT to determine the presence of ICH. Both were judged by two experienced neurointerventionists, and in the case of contrasting opinions, consensus was reached through consultation.

### 2.5. Statistical Analysis

Characteristics are summarized as proportions for categorical variables and mean ± SD or median (25–75th percentile) for quantitative variables, as appropriate. Fisher’s exact test was used to compare the dichotomous variables between groups, while the independent samples *t*-test or Mann-Whitney U test was used for the continuous variables. The effect of tirofiban on clinical outcomes in different subgroups was evaluated using logistic regression models, results were expressed as relative risk odds ratios (ORs) and 95% confidence intervals (CIs). *p* values of < 0.05 were considered statistically significant. All reported *p* values were 2-sided. All statistical analyses were performed using IBM SPSS Statistics for Windows version 22 and R language 4.0.5.

## 3. Results

### 3.1. Demographic and Clinical Characteristics

**Figure 1:**
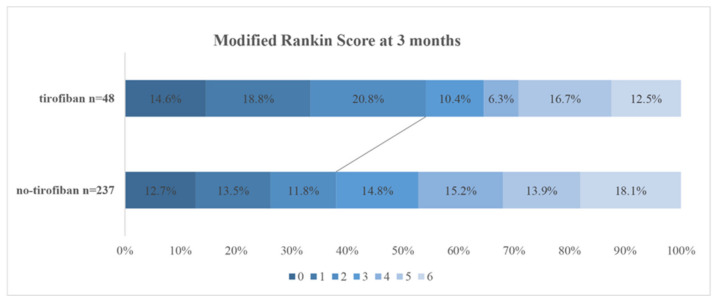
Distribution of mRS at 3 months categories in the overall patients. The lines indicate differences in favorable outcome (mRS 0–2) between groups. mRS, modified Rankin Scale.

A total of 285 patients included. The median age was 71 (63, 78) years, 61.7% were female, and 48 were treated with tirofiban and 237 were not. Baseline characteristics are shown in Table 1. Patients treated with tirofiban were more likely to develop large-artery atherosclerotic (LAA) stroke (70.8% vs. 47.6%, *p* = 0.002) and less likelywithatrial fibrillation (29.1% vs. 62.0%, *p* = 0.000). Nevertheless, the rate of recanalization was not significantly different between the two groups. (89.5% vs. 92.4%, *p* = 0.513). After 3 months of follow-up, 116 (40.7%) patients achieved favorable outcomes, and 49 (17.1%) patients died. A favorable outcome was observed significantly more often in patients treated with tirofiban (54.1% vs. 37.9%, *p* = 0.037) Figure 1. There was no significant difference in ICH (*p* = 0.980), sICH (*p* = 0.924) or mortality (*p* = 0.345) between the two groups.

### 3.2. Favorable Functional Outcome

Stepwise regression analysis was used to determine the factors that were independently associated with favorable functional outcome. The results demonstrated that age, NIHSS score, ASPECTS score, administration of tirofiban, and achievement of TICI2b-3 were associated with favorable outcomes (*p* < 0.1), with all variance inflation factors (VIFs) < 5. Considering existing studies and clinical experience, stroke etiology, OTP time and OTR time were also included in the multivariate logistic regression models (Table 2). After adjusting for confounding factors, the administration of tirofiban was associated with a significant increase in the rate of favorable outcomes (aOR 2.033, 95% CI, 1.002~4.123, *p* = 0.043). Lower age and lower NIHSS score were also associated with higher odds of favorable outcome (*p* = 0.000). Within the tirofiban group separately, the outcome of 48 patients was also affected by age and NIHSS score (*p* = 0.000), which was in line with the entire population (Table S1, See Supplementary Materials).

### 3.3. Subgroup Analysis

**Figure 2:**
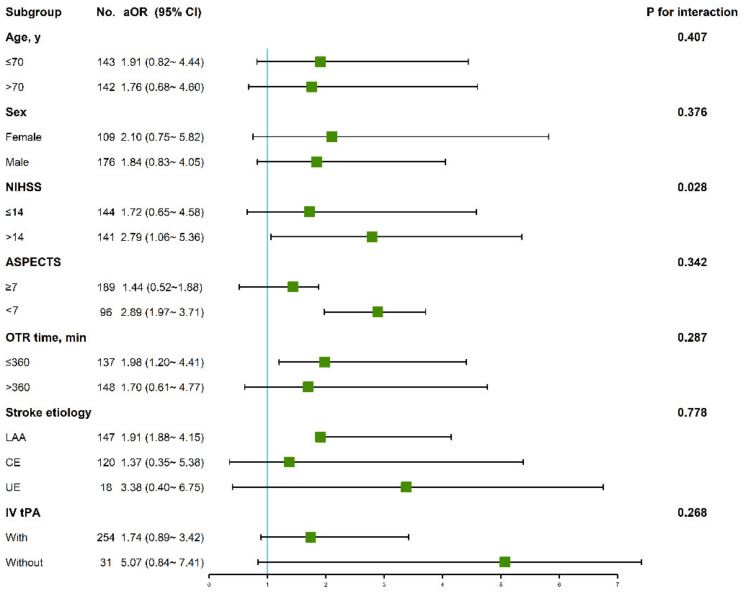
Forest plot aOR, adjusted odds ratios of tirofiban for favorable outcome. Adjusted for age, NIHSS, ASPECTS, stroke etiology, administration of tirofiban, OTP time, OTR time, TICI 2b-3. NIHSS, National Institutes of Health Stroke Scale; ASPECTS, Alberta Stroke Program Early Computerized Tomography Score; CI, confidence interval; OTP, time from onset to groin puncture; OTR time from onset to recanalization; TICI, Thrombolysis in Cerebral Infarction grading.

**Figure 3:**
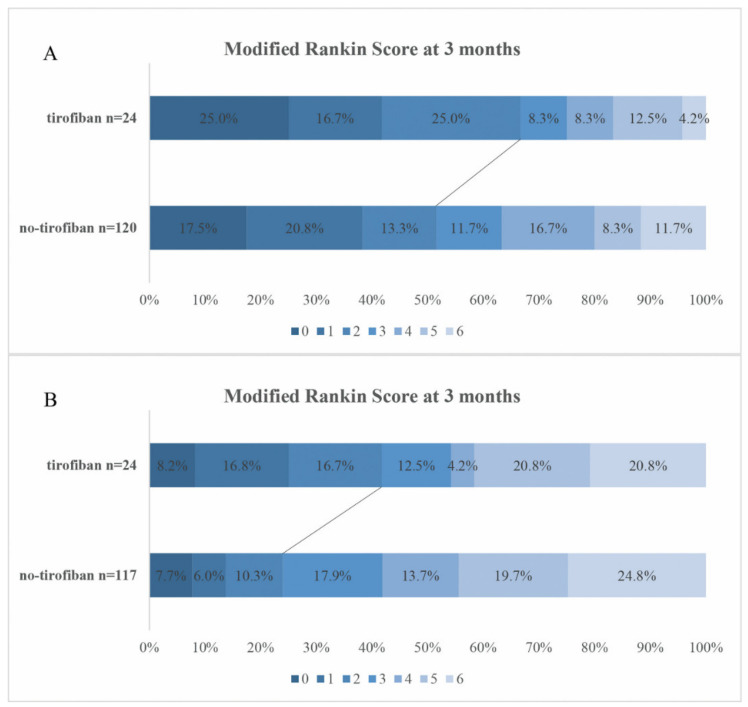
Distribution of mRS at 3 months categories in the patients with NIHSS ≤ 14 (**A**) and NIHSS > 14 (**B**). The lines indicate differences in favorable outcome (mRS 0–2) between groups. mRS, modified Rankin Scale.

The heterogeneity of the effect of tirofiban on the favorable clinical outcome by baseline characteristics and severity of stroke was assessed in subgroups analysis in Figure 2. There was significant heterogeneity across the subgroups according to the NIHSS score (*p* for interaction = 0.028), suggesting that the effect of tirofiban was modified by the NIHSS score. In the 141 patients with a NIHSS score exceeding 14, tirofiban was associated with an increase in the rate of favorable outcome (aOR 2.79, 95% CI 1.46~7.36) but not in the patients with a NIHSS score of 14 or below (aOR 1.72, 95% CI 0.65~4.58). The mRS distribution stratified by NIHSS score is shown in Figure 3. Although there was no heterogeneity in the effect of tirofiban on favorable outcomes with respect to age, sex, ASPECTS, OTP time, use of t-PA and stroke etiology (*p* for interaction >0.05), tirofiban was associated with outcomes in the subgroup of time from onset to puncture ≤360 (aOR 1.91, 95% CI 1.20~4.41) and stroke due to LAA (aOR 1.91, 95% CI 1.88~4.15).

### 3.4. Safety Outcome

Tirofiban was not associated with an increased risk of adverse outcomes, including ICH (OR 1.07, 95% CI 0.55~2.06), sICH (OR 1.16, 95% CI 0.52~2.56) and mortality (OR 0.78, 95% CI 0.30~2.02). However, older age and higher NIHSS were identified as risk factors for hemorrhage and mortality (*p* < 0.05) (Table S2). In both the NIHSS ≤ 14 and NIHSS > 14 (*n* = 144) groups, tirofiban was not associated with an increased risk of ICH, sICH or mortality, and all *p* for interaction was > 0.05 (Table 3).

## 4. Discussion

MT has been demonstrated to be able to revascularize occluded intracranial arteries and accelerate the recovery of AIS patients. However, it inevitably fails to achieve recanalization in approximately 20% of AIS patients [11], and early re-occlusion may occur, especially when endothelial damage occurs [12]. Tirofiban, a glycoprotein IIb/IIIa antagonist with a short half-life, is suggested to avert the risks of thromboembolic complications and improve the reperfusion status of the microvasculature [13,14,15], especially for patients with severe in situ atherosclerotic stenosis and permanent stenting. However, evidence about patient selection and the treatment strategy for the different subgroups remains ambiguous.

In our study, the post-hoc subgroup analysis demonstrated that tirofiban was more associated with an increasingly favorable outcome in patients with NIHSS scores > 14rather than those whose NIHSS < 14. It may indicate that tirofiban was more effective in patients with severe stroke. Although the underlying mechanism is unknown, a possible explanation is that patients with higher NIHSS are at an inherent higher risk for failure of recanalization [16]. First, higher NIHSS scores were significantly correlated with poor collateral circulation, which suggests that blood flow is difficult to restore to the level of TICI 2B-3 [17,18]. Nevertheless, robust collateral circulation is important for the prevention of thrombus augmentation, dissolution of the fragmented thrombus and increasing the concentration of local thrombolytic drugs [19]. Second, patients with higher NIHSS scores are often accompanied by occlusion of proximal large vessels with larger volume clot [20,21,22], which may be more difficult to remove. Therefore, we assume that tirofiban can give exert optimal advantages in patients with moderate and severe stroke who may benefit more from the increasing recanalization rate [23,24]. In our study, the recanalization rate in the tirofiban group was lower than that in the no-tirofibangroup (89.6% vs. 92.4%, *p* = 0.521), which is consistent with the results from previous studies wherein tirofiban did not increase the recanalization rate [7]. This may be explained by the fact that tirofiban was prone to be used in patients with a high possibility of reocclusion, and not all of them were recanalized after tirofiban or other rescue strategies, such as intra-arterial thrombolysis and angioplasty. The recanalization rate defined as TICI 2b-3 only showed the final outcome, and the potential benefit of promoting recanalization needs further study.

Based on the findings of our study, the effect of tirofiban was not significantly modified by age, sex, ASPECTS, time from onset to puncture, use of t-PA or stroke etiology, which provided further support for the benefit of tirofiban in AIS patients, regardless of the baseline characteristics. Previous studies suggest that tirofiban is more effective in LAA patients than in CE patients [25]. Our results also demonstrated that the association between tirofiban and clinical outcome was significant in LAA patients (aOR 1.91, 95% CI 1.88~4.15) but not in the other two groups. This finding is expected when atherosclerotic occlusion may complicate reperfusion, and re-occlusion can easily occur even after successful recanalization by in situ formation of microthrombus, conditions more likely to require antiplatelet therapy such as tirofiban. The crucial effect of time has been emphasized in relation to endovascular therapies for AIS patients [26]. Both OTP time and OTR time influenced the outcome, and we performed subgroup analysis according to the OTP time, which occurs before MT and may be considered when operators make decisions about the administration of tirofiban [27]. However, our study did not show heterogeneity in the subgroups according to OTP time, and large-sample size studies might be needed for further verification. Additionally, it should be taken into account that in the retrospective study, the OTP time of patients was based on the medical records derived from statements of patients and their family members, which was likely inaccurate and unreliable.

In safety-related analyses, tirofiban also did not increase the risk of bleeding in either the overall group or the subgroup, which was consistent with most studies and meta-analyses [28]. However, Lars Keller et al. [9] showed that additional treatment with tirofiban was associated with an increased risk of fatal intracerebral hemorrhage. Possible underlying causes of this result are that the patients were treated before 2011 with relatively unadvanced thrombectomy devices which achieved a low recanalization rate (61.1%). Given that tirofiban has a short half-life and rapid drug metabolism, tirofiban may be safe for selected patients.

Consider the inevitable bias and false positive in the post-hoc subgroup analysis, the results need to be interpreted more carefully, and exact conclusions need to be further verified in prospective studies. Our results further support the effect and safety of tirofiban in AIS patients, and NIHSS score could be taken into consideration as appropriate when making a decision on the administration of tirofiban.

Our study has several limitations. First, this was a retrospective study and has a risk of selection bias. We adjusted for potential confounders through the multivariable logistic regression model and obtained the same results. Second, these post hoc subgroup analyses may lack power, and the findings may have been accidental. A more detailed subgroup analysis according to NIHSS score was not performed due to the small sample size. Finally, other prognostic factors, such as collateral status, specific location of infarction, and remaining penumbral tissue, were not included in this study, which was potential confounders. The underlying mechanisms of tirofiban in AIS need further investigation, and prospective clinical trials are still needed to provide higher-level evidence.

## 5. Conclusions

The administration of tirofiban was associated with an increased rate of favorable outcomes in patients undergoing MT with severe ischemic stroke, and further studies are needed to confirm this finding.

## Figures and Tables

**Table 1:** Patient characteristics and clinical outcomes.

	No Tirofiban *N* = 237	Tirofiban *N* = 48	Overall *N* = 285	*p* Value
**Age, y**	71 (64, 79)	69 (58, 76)	71 (63, 78)	0.165
**Female, *n* (%)**	146 (61.6)	30 (62.5)	176 (61.7)	0.907
**Stroke etiology, *n* (%)**				0.002
**LAA**	113 (47.6)	34 (70.8)	147 (51.5)	
**CE**	111 (46.8)	9 (18.7)	120 (42.1)	
**UE**	13 (5.5)	5 (10.4)	18 (6.3)	
**NIHSS, median (IQR)**	14 (11, 18)	14 (10, 17)	14 (11,18)	0.173
**ASPECTS, median (IQR)**	8 (7–9)	8 (7–9)	8 (7–9)	0.788
**Systolic BP, mmHg**	142.5 ± 17.7	147.4 ± 21.4	143.4 ± 18.5	0.091
**Glucose, mmol/L**	8.6 ± 23.1	6.9 ± 1.6	8.3 ± 21.0	0.605
**Medical History**				
**Atrial fibrillation, *n* (%)**	147 (62.0)	14 (29.1)	161 (56.4)	0.000 *
**Hyperlipidemia, *n* (%)**	5 (2.1)	1 (2.0)	6 (2.11)	0.991
**Hypertension, *n* (%)**	154 (64.9)	29 (60.4)	183 (64.2)	0.548
**Diabetes mellitus, *n* (%)**	38 (16.0)	6 (12.5)	44 (15.4)	0.537
**Previous stroke, *n* (%)**	39 (16.4)	7 (14.5)	46 (16.1)	0.748
**Pre-antiplatelet, *n* (%)**	34 (14.3)	7 (14.5)	41 (14.3)	0.966
**Pre-anticoagulation, *n* (%)**	23 (9.70)	1 (2.0)	24 (8.4)	0.083
**Smoker, *n* (%)**	36 (15.1)	9 (18.7)	45 (15.7)	0.537
**Procedural Variables**				
**t-PA, *n* (%)**	209 (88.2)	45 (93.7)	254 (89.1)	0.351
**OTP time, min**	305 (190, 365)	350 (245, 420)	310 (215, 390)	0.034 *
**OTR time, min**	360 (285, 450)	455 (355, 532)	375 (290, 460)	0.001 *
**TICI 2b-3, *n* (%)**	219 (92.4)	43 (89.5)	262 (91.9)	0.513
**Retrieval times ≥ 3, *n* (%)**	8 (3.4)	5 (10.4)	13 (4.6)	0.045 *
**Rescue therapy** **#, *n* (%)**	27 (11.4)	11 (22.9)	38 (13.3)	0.024 *
**Clinical Outcome**				
**Favorable outcome, *n* (%)**	90 (37.9)	26 (54.1)	116 (40.7)	0.037 *
**sICH, *n* (%)**	49 (20.7)	10 (20.8)	59 (20.7)	0.980
**ICH, *n* (%)**	97 (40.9)	20 (41.6)	117 (41.0)	0.924
**Mortality at 3 m, *n* (%)**	43 (18.1)	6 (12.5)	49 (17.1)	0.345

LAA, Large artery atherosclerosis; CE, Cardio embolism; UE, Unclear etiology; NIHSS, National Institutes of Health Stroke Scale; BP, blood pressure; t-PA, tissue plasminogen activator; OTP, time from onset to groin puncture; OTR, time from onset to reperfusion; TICI: Thrombolysis in Cerebral Infarction grading. ICH, intracranial hemorrhage; sICH, symptomatic intracranial hemorrhage; Values are *n* (%), mean ± SD, median (interquartile range); # Rescue therapy included balloon angioplasty and permanent stenting; * statistically significant.

**Table 2:** Multivariate regression analysis for favorable outcome.

	OR (95% CI)	*p* Value	aOR # (95% CI)	*p* Value
Age	0.958 (0.938~0.979)	0.000 *	0.953 (0.930~0.977)	0.000 *
Stroke etiology	0.665 (0.352~1.167)	0.424	0.875 (0.662~1.158)	0.353
NIHSS	0.873 (0.828~0.921)	0.000 *	0.877 (0.829~0.928)	0.000 *
ASPECTS	0.842 (0.723~0.839)	0.024 *	0.738 (0.796~0.916)	0.039 *
Use of tirofiban	1.930 (1.033~3.608)	0.039 *	2.033 (1.002~4.123)	0.043 *
OTP time	0.999 (0.998~1.002)	0.892	0.993 (0.829~0.928)	0.205
OTR time	0.993 (0.938~0.979)	0.032 *	0.993 (0.982~0.993)	0.043 *
TICI 2b-3	4.539 (1.938~3.979)	0.027 *	3.167 (0.997~1.001)	0.056

aOR, adjusted odds ratio; CI, confidence interval; NIHSS, National Institutes of Health Stroke Scale; ASPECTS, Alberta Stroke Program Early Computed Tomography Score; OTP, time from onset to groin puncture; OTR time from onset to recanalization; TICI, Thrombolysis in Cerebral Infarction grading; * statistically significant; # model adjusted by age, NIHSS, ASPECTS, stroke etiology, administration of tirofiban, OTP time, OTR time, TICI 2b-3.

**Table 3:** Multivariate regression analysis of safety outcome in subgroups according to NIHSS.

	NIHSS ≤ 14 (*n* = 141)		NIHSS > 14 (*n* = 144)	
	aOR (95% CI)	*p* Value	aOR (95% CI)	*p* Value
ICH	1.047 (0.396~2.770)	0.926	1.812 (0.311~2.117)	0.670
sICH	1.779 (0.530~1.980)	0.717	1.024 (0.396~2.770)	0.944
Death	0.530 (0.060~4.499)	0.553	0.732 (0.495~1.055)	0.092

aOR, adjusted odds ratio; CI, confidence interval; NIHSS, National Institutes of Health Stroke Scale model adjusted by age, NIHSS, ASPECTS, stroke etiology, administration of tirofiban, OTP time, OTR time, TICI 2b-3.

## Data Availability

The datasets used and/or analyzed during the present study are available from the corresponding author on reasonable request.

## Supplementary Materials

The following supporting information can be downloaded at: https://www.mdpi.com/article/10.3390/jcdd9110408/s1, Table S1: Multivariate regression analysis for favorable outcome in the patients with tirofiban; Table S2: Multivariate Regression Analysis for safety outcome.
