# Supplementary material for: Safety and Efficacy of Tirofiban in Severe Ischemic Stroke Patients Undergoing Mechanical Thrombectomy
Source: J Cardiovasc Dev Dis. 2022 Nov 21;9(11):408. doi: 10.3390/jcdd9110408 (PMC9699197; doi:10.3390/jcdd9110408)
Supplement: Supplementary file 1 [file jcdd-09-00408-s001.zip › jcdd-1923407-supplementary.pdf]

Supplemental Table S1 Multivariate regression analysis for favorable outcome in the patients with tirofiban

	OR (95% CI)	P	aOR (95% CI)	P
Age	0.977 (0.922~0.935)	0.026*	0.966 (0.915~0.980)	0.034*
Stroke etiology	0.909 (0.602~1.770)	0.909	0.743 (0.543~1.987)	0.548
NIHSS	0.824 (0.705~0.963)	0.015*	0.853 (0.745~0.977)	0.022*
ASPECTS	0.823 (0.614~0.834)	0.034*	0.745 (0.874~0.934)	0.027*
OTP time	1.005 (0.990~1.020)	0.500	1.000 (0.994~1.005)	0.886
OTR time	0.992 (0.970~0.991)	0.030*	0.966 (0.984~0.997)	0.043*
TICI 2b-3	2.475 (0.753~1.386)	0.067	1.859 (0.672~1.573)	0.173

aOR, adjusted odds ratio; model adjusted by age, NIHSS, ASPECTS, stroke etiology, OTP time, OTR time, TICI 2b-3. CI, confidence interval; NIHSS, National Institutes of Health Stroke Scale; ASPECTS, Alberta Stroke Program Early Computed Tomography Score; OTP, time from onset to groin puncture; OTR time from onset to recanalization; TICI, Thrombolysis in Cerebral Infarction grading; * statistically significant;

Supplemental Table S2 Multivariate Regression Analysis for safety outcome

	ICH		SICH		Death	
	OR (95% CI)	P	OR (95% CI)	P	OR (95% CI)	P
age	1.01 (0.99~ 1.03)	0.065	1.03 (1.00~ 1.06)	0.030*	1.04 (1.01~ 1.08)	0.008*
NIHSS	1.05 (1.01~ 1.10)	0.010*	1.07 (1.02~ 1.12)	0.002*	1.09 (1.03~ 1.14)	0.001*
ASPECTS	1.02 (0.94 ~1.44)	0.052	1.03 (1.04~ 1.22)	0.035*	1.08 (1.04~1.09)	0.014*
tirofiban	1.07 (0.55~ 2.06)	0.835	1.16 (0.52~ 2.56)	0.712	0.78 (0.30~ 2.02)	0.611
OTP time	1.00 (1.00~ 1.00)	0.510	1.00 (1.00~ 1.00)	0.265	1.00 (1.00~ 1.00)	0.741
OTR time	1.00 (1.00~ 1.00)	0.738	1.00 (1.00~ 1.00)	0.453	1.00 (1.00~ 1.00)	0.824

OR, odds ratio; CI, confidence interval; NIHSS, National Institutes of Health Stroke Scale; ASPECTS, Alberta Stroke Program Early Computed Tomography Score; OTP, time from onset to groin puncture; OTR time from onset to recanalization; TICI, Thrombolysis in Cerebral Infarction grading; * statistically significant;
